# A Mixed-Methods Feasibility Pilot Study of Medimon for High School Endocrine Education

**DOI:** 10.3390/mti10080084

**Published:** 2026-08-08

**Authors:** Sebastian Svoboda-Powell, Shane Mitchell, Ciara Bordeaux, Emma Ferguson, Joscelyn Coad, Alexandra Silalahi, Cameron Bowman, Jessi Holte, Patryk Kosmulski, Tyler Bland

**Affiliations:** 1 School of Medicine, University of Washington, Seattle, WA 98195, USA; 2 WWAMI Medical Education Department, University of Idaho, Moscow, ID 83844, USA; 3 Panacea Interactive LLC, Moscow, ID 83844, USA

**Keywords:** game-based learning, endocrine physiology, visual mnemonics, artificial intelligence, high school education

## Abstract

Medimon is an educational role-playing game that integrates visual mnemonics, collectible creatures, disease states, treatment items, and artificial intelligence-powered non-player characters (AI-NPCs) to teach biomedical concepts. This single-arm mixed-methods feasibility pilot examined recruitment, gameplay uptake and progression, player experience, descriptive knowledge outcomes, and AI-NPC interactions during the Medimon endocrine level among high school students. Thirty students consented; 10 generated gameplay data, six completed paired 12-item pretests and posttests, and eight completed a PXI-style survey. Students played independently using a Steam-based build and encountered thyroid, pancreas, and adrenal educational content through exploration, quests, battles, and Medimon collection. Mean paired knowledge scores increased from 19.4% at pretest to 44.4% at posttest, although the change was not statistically significant in this small exploratory sample (*p* = 0.156). Audiovisual appeal, curiosity, and enjoyment were rated favorably, whereas Progress Feedback was the lowest-rated player-experience domain. Students who completed the posttest demonstrated greater gameplay duration, exploration, quest completion, and Medimon exposure than non-completers. Thematic analysis identified a mismatch between player expectations and AI-NPC scope, including fabricated quests, spatial directions, and game mechanics that introduced unreliable guidance into the educational environment. The study identified barriers related to gameplay uptake, progression feedback, posttest completion, and AI-NPC reliability that should be addressed before the educational efficacy of Medimon is evaluated in a larger controlled study.

## 1. Introduction

Physiology education requires learners to integrate relationships across multiple levels of organization, including cells, tissues, organs, signaling molecules, disease states, and therapeutic interventions [1–3]. Rather than learning isolated factual associations, students must construct explanatory models that connect normal structure and function with pathophysiology and treatment [3–5]. This integrated and dynamic nature of the discipline has been identified as a major reason physiology is difficult for learners, particularly when students have limited opportunities to visualize invisible biological processes or apply concepts in meaningful contexts [6]. These challenges are relevant before students enter health-professions programs. Strengthening foundational biological understanding during high school may help students develop the conceptual frameworks needed to reason about later coursework in anatomy, physiology, pathology, pharmacology, and medicine [7,8].

Active learning approaches can improve performance in science, technology, engineering, and mathematics education, particularly when students must retrieve, apply, discuss, or use knowledge rather than passively receive information [8–10]. Digital game-based learning is one approach for creating such opportunities [11,12]. Well-designed games can combine goals, narrative context, exploration, repeated practice, feedback, challenge, player agency, and consequential decision-making within a unified environment [13,14]. In contrast to a conventional quiz or isolated digital learning activity, a game can position academic content as part of a larger system that the learner must explore and use to progress.

Evidence syntheses suggest that digital games can support learning, although outcomes depend substantially on implementation and design quality. A systematic review and meta-analysis of K–16 digital games found that game-based conditions were associated with improved learning relative to nongame conditions and that design features, including game mechanics and narrative elements, moderated outcomes [15]. Similarly, a meta-analysis of serious games reported favorable cognitive learning effects compared with conventional instruction, particularly when games were supplemented with other instructional elements and delivered across multiple sessions [16]. Broader reviews have identified knowledge acquisition, engagement, and motivation as frequent outcomes of computer games and serious games, while also noting substantial variability in study quality, game type, learning context, and outcome measurement [17]. Meta-analytic evidence for gamification likewise suggests small positive effects on cognitive, motivational, and behavioral outcomes, but emphasizes that benefits are not automatic and may be sensitive to the specific design elements used [18,19].

Game-based approaches may be particularly useful for biomedical learning because they can transform abstract concepts into visible, interactive systems. In health-professions education, serious games and gamification have been associated with learner satisfaction and, in some studies, improved knowledge or skills; however, the certainty of evidence has often been limited by heterogeneous interventions, small samples, and variable study quality [20–22]. These limitations highlight the importance of evaluating not only whether a serious game is enjoyable, but also whether its educational content, learning objectives, game mechanics, and outcome measures are deliberately aligned. Frameworks for serious-game design emphasize the need to connect pedagogical goals with mechanics such as exploration, collection, feedback, challenge, and progression rather than simply adding superficial game features to conventional instructional content [23,24].

One promising strategy for making biomedical content more memorable is the purposeful use of visual and verbal mnemonics [25–27]. Dual-coding theory proposes that learning can be supported when information is represented through complementary verbal and nonverbal systems [28,29]. Multimedia-learning theory similarly emphasizes that learners construct understanding from coordinated visual and auditory/verbal representations, provided that instructional materials guide attention to relevant information and avoid unnecessary cognitive load [30,31]. In science education, visual representation and drawing can support explanation, reasoning, and the integration of complex relationships that may be difficult to communicate through text alone [32]. Experimental work in other learning contexts has also shown that visual mnemonics can facilitate learning by linking unfamiliar material to meaningful visual features and elaborative associations [33].

A role-playing game may offer a practical structure for combining these principles [34,35]. Characters, settings, items, quests, and battle mechanics can repeatedly connect physiological concepts with visible representations and meaningful player actions. For example, a healthy cell-type character can be paired with a disease-associated variant and a treatment item, while the surrounding environment can reinforce the organ’s normal function through architecture, objects, narrative events, and visual metaphors. Repeated encounters with these linked representations may create opportunities for retrieval, elaboration, and application that extend beyond identifying a term on a conventional assessment. However, the educational value of such systems depends on whether students meaningfully interact with the content rather than merely encounter it during gameplay [36,37].

Artificial intelligence (AI)-enabled conversational characters may extend these opportunities by allowing players to ask questions without leaving the game environment. Research on intelligent tutoring systems suggests that adaptive computer-based tutoring can improve learning outcomes relative to conventional instruction, although effectiveness varies by implementation and context [38–40]. More recently, large language models have created new possibilities for open-ended educational dialogue, including explanation, individualized practice, and domain-specific conversational support [41]. At the same time, educational deployment of large language models requires attention to factual accuracy, bias, privacy, transparency, learner overreliance, and appropriate human oversight [41,42]. Within a game-based educational environment, AI-powered non-player characters (AI-NPCs) may provide a contextually situated way for learners to ask questions of characters associated with a specific biomedical domain [43]. Non-player characters (NPCs) are computer-controlled characters that provide dialogue, narrative information, quests, or other gameplay functions without being directly controlled by the player. Conventional NPCs generally rely on predetermined dialogue and behavior, whereas AI-enabled NPCs can generate open-ended responses to player input. AI-NPCs may extend the educational role of conventional NPCs by responding to unanticipated questions without requiring learners to leave the game environment. This creates opportunities for situated explanation and dialogue but also introduces risks related to factual accuracy, role ambiguity, and inconsistent behavior [41,44,45]. Empirical evidence regarding AI-NPCs in educational games remains limited, and the present study was not designed to determine whether the visual realism of an NPC affects its educational effectiveness.

Medimon is an educational role-playing video game originally designed to teach biomedical concepts to health-professions learners through collectible creature characters, environmental visual mnemonics, disease states, treatment items, quests, and strategic battles [46,47]. The endocrine level of Medimon focuses on foundational thyroid, pancreas, and adrenal physiology; selected disease states; and associated treatments. Educational concepts are embedded in organ-family environments, including Shield Electric for thyroid physiology, Digestum Aquarium for pancreas physiology, and Thirsty’s Haunted Mansion for adrenal physiology. Players can encounter, battle, capture, and add Medimon to an active team, thereby interacting with educational material at varying levels of depth. The level also includes three AI-NPCs intended to answer organ-specific scientific and medical questions related to the thyroid, pancreas, and adrenal families.

The novelty of the present study lies in the integration of newly developed endocrine content, a high school learner population, multimodal role-playing gameplay, embedded generative AI-NPCs, gameplay telemetry, and authentic student–AI interaction analysis. The feasibility design limits conclusions regarding educational efficacy but does not diminish the distinct implementation and interaction-design questions evaluated.

The present study evaluated the feasibility and exploratory educational outcomes of this Medimon endocrine level implementation among high school students. Specifically, the study examined student participation, home-based gameplay progression, gameplay telemetry, completion of endocrine level knowledge assessments, descriptive pretest-to-posttest change, player experience, and use of the embedded AI-NPCs. Given the early-stage, single-arm design and small sample, the purpose was not to establish educational efficacy, but to characterize implementation, generate preliminary estimates for future study design, and identify barriers and development priorities.

## 2. Materials and Methods

### 2.1. Study Design and Ethical Approval

This study used a single-arm, mixed-methods pilot design to evaluate the feasibility, gameplay progression, preliminary knowledge outcomes, and player experience associated with the endocrine level content of Medimon, an educational role-playing video game. The study was approved by the University of Idaho Institutional Review Board (IRB Protocol 25–144). Signed parental consent and student assent were obtained before participation.

The study focused exclusively on endocrine level gameplay content. Although the study build contained additional game levels, later-level data were not included in the present analysis because the study was designed and analyzed around the endocrine level educational experience. Within the NIH STTR Phase I project supporting this work, the study was designed as an early feasibility and design-refinement evaluation rather than a definitive test of educational efficacy.

### 2.2. Participants and Recruitment

Participants were students in grades 9 through 12 recruited from two public high schools in the inland northwestern United States: Moscow High School in Moscow, Idaho, and Clarkston High School in Clarkston, Washington. Publicly available school-level demographic data indicate that both schools serve predominantly White student populations, with approximately equal proportions of male and female students [48,49]. Participant-level demographics were not collected. Before the study, the principal investigator visited each school for 1 day and introduced the project during science-related class periods. Students were invited to participate if they were enrolled in one of the participating classes and returned signed parental consent and student assent forms. No additional eligibility criteria were applied.

Thirty students provided consent and assent. Of these, 10 students generated gameplay data during the study period and were included in gameplay, telemetry, and knowledge-assessment analyses. The anonymous post-play survey was completed by eight students. Students received a $20 incentive upon completing all requested study components: gameplay, the post-study survey or in-game posttest, and the player-experience survey.

### 2.3. Study Procedures and Endocrine Level Game Environment

The study was conducted from 18 May through 1 June 2026. Students received a Steam activation key and written instructions describing how to install Steam, activate the game, and launch Medimon. Students were also provided a YouTube how-to-play video [50] and a PDF instruction packet describing the game’s primary goals, quest progression, Medimon collection and battle mechanics, AI-powered non-player characters (AI-NPCs), and safe AI-use expectations (Supplemental Methods S1).

Students independently played the game at home using the Steam platform [51]. The study used Medimon Build 23281743, released on 17 May 2026, and available for both Windows and macOS. Students were encouraged, but not required, to play for at least 1 h during the study period.

At the start of the endocrine level, all students began in Sylva Forest and selected one of three available starter Medimon: Cholangiocyte, Neuron, or B-Cell. Eight students selected Cholangiocyte, two selected Neuron, and no students selected B-Cell. The endocrine level map contained three principal organ-family learning environments: Shield Electric, representing the thyroid family; Digestum Aquarium, representing the pancreas family; and Thirsty’s Haunted Mansion, representing the adrenal family (Figure 1). The map also included noneducational navigational and environmental areas needed for gameplay progression; these were not the primary focus of the present analysis.

Students completed quests, explored locations, encountered wild Medimon, battled diseased Medimon, collected treatment items, and could add captured Medimon to an active battle team of up to six members. Treatment items were distributed as pickups within the game world and were also available for purchase from the in-game hospital pharmacy, Sacred Lake Medical Center. Educational content was embedded in the visual design of the environments and Medimon, the disease-treatment interactions, item descriptions, character dialogue, and quest structure.

### 2.4. Endocrine Level Educational Design and Visual Mnemonics

The endocrine level was designed to introduce foundational thyroid, pancreatic, and adrenal physiology; basic pathophysiology of related disease states; and treatment associations. Rather than presenting these concepts primarily through didactic text, the game embedded educational information in the appearance, names, evolutionary relationships, treatment items, environmental architecture, and story-based quests of the Medimon world. Interaction with the endocrine level combined visual character and environmental representations, written dialogue and item descriptions, spatial exploration, battle and collection actions, and open-ended AI conversation. The present study evaluated this integrated multimodal system as a whole and was not designed to isolate the contribution of any individual modality. The descriptions below are provided to define the intervention and explain how endocrine concepts were represented across visual, textual, spatial, mechanical, and conversational modalities rather than to document every game asset.

The endocrine level was designed primarily to support initial recognition and association rather than comprehensive conceptual change. Visual and verbal mnemonics introduced relationships among healthy physiology, disease states, and treatments; quests and treatment-item use required players to apply these associations within the game; and battles, captures, and repeated environmental encounters provided additional opportunities for retrieval and reinforcement. AI-NPCs offered optional organ-specific explanations. The study build did not include adaptive misconception diagnosis or individualized corrective feedback outside of negative battle effects for incorrect treatment item usage on a diseased Medimon, and formal endocrine feedback loops were outside the stated learning objectives.

Each family included three healthy Medimon, three disease-associated Medimon, and three treatment items corresponding to the associated physiological or pathological concepts (Figures 2, 4 and 6). Each organ-family area used a distinct building and environmental design to represent underlying anatomy and physiology (Figures 3, 5 and 7). Players could encounter and battle Medimon, capture selected Medimon after battle, and add captured Medimon to their active team. Thus, interaction with educational content could occur at multiple levels, including environmental exploration, observation of Medimon, battle encounters, capture, team use, item collection, and disease-treatment gameplay.

#### 2.4.1. Thyroid Family and Shield Electric

Thyroid-family and related content (Figure 2) was located in Shield Electric, an environment designed as a hydroelectric dam and power-generation facility to represent the energy-related role of thyroid hormone (Figure 3). The exterior included a dam, power lines, and a central industrial building. The interior contained large follicularturbine structures shaped to resemble thyroid glands. Water containers labeled “I” entered the turbines as a visual mnemonic for iodine, whereas T3 and T4 batteries emerged from the turbines to represent thyroid hormone production. Dump trucks labeled “TG” represented thyroglobulin and were depicted transporting the T3 and T4 battery objects through the facility.

A secondary visual mnemonic represented parafollicular C-cell function. A milk vending machine, representing calcium through the game’s “cowcium” visual metaphor, emptied milk through a drain beneath a sign reading “Milk Cleanout Station” with a prominent “C.” This feature was designed to represent the role of parafollicular C-cells and calcitonin in decreasing serum calcium. Thyroid-related disease-treatment interactions were incorporated into quest progression; for example, an early quest involved using a T4-related treatment item against a rogue Hashimoto Disease Medimon.

#### 2.4.2. Pancreas Family and Digestum Aquarium

The pancreas family and related content (Figure 4) were located in Digestum Aquarium, a fish-themed environment reflecting the visual design of the pancreas-family Medimon and the digestive functions of the pancreas (Figure 5). The building’s exterior was designed with a stomach-like form to connect the pancreas’s role in digestion, whereas its interior layout included a rounded food-court lobby representing the pancreatic head and an extended hallway, termed the Islet Gallery, representing the pancreatic body and tail. The Islet Gallery contained column-like aquarium tanks housing Alpha Cell and Beta Cell Medimon to represent the islets of Langerhans.

Digestum Aquarium also included a basement level with a damaged, sunken-ship visual motif and a donut stand. These environmental elements represented pancreatic breakdown and autodigestion in pancreatitis and dysregulated glucose metabolism in diabetes, respectively. Together, the building layout, aquarium setting, cell-specific displays, disease-associated environmental cues, and treatment items were intended to reinforce basic pancreatic anatomy, islet-cell physiology, diabetes, pancreatitis, and related treatment concepts.

#### 2.4.3. Adrenal Family and Thirsty’s Haunted Mansion

The adrenal family and related content (Figure 6) were located in Thirsty’s Haunted Mansion, a multilevel environment combining haunted-mansion, water-park, stress, and fight-or-flight themes (Figure 7). The building descended through several levels to represent progression from the outer to inner layers of the adrenal gland. The overall snake-like visual design paralleled the serpent-inspired appearance of the adrenal-family Medimon.

The upper level, Golden Rapids Water Park, represented the zona glomerulosa and aldosterone. Its “Golden” title served as a mnemonic for “glomerulosa”, whereas its water-park design represented aldosterone-mediated water reabsorption. A mining and mineral theme represented “mineral” corticoid activity, and salt water represented aldosterone-mediated sodium reabsorption. Shell-shaped side tables referenced the shell-associated Kidney Medimon, an octopus-like character, to reinforce the renal site of aldosterone action.

The next level used haunted-mansion imagery to represent cortisol and physiological stress. This environment included intentionally stressful visual scenes, such as depictions of interpersonal conflict, job loss, financial loss, illness, negative news, and poor examination performance. A subsequent middle-school gym environment represented puberty and the contribution of adrenal androgens to pubertal development. The final level, Fight or Flight Club, represented adrenal-medullary activation and sympathetic fight-or-flight physiology. The associated building image shown in Figure 7 depicts Golden Rapids Water Park, with an inset of the building exterior.

The endocrine level educational design emphasized healthy physiology, basic disease associations, and treatments. Formal endocrine feedback-loop instruction was not a primary learning objective in this level.

### 2.5. AI-Powered Non-Player Characters

Three AI-powered NPCs were available within the endocrine level: Cooper, representing the thyroid family; Josea, representing the pancreas family; and Addy Stone, representing the adrenal family (Figure 8). Each character was represented by a portrait image and an in-game sprite. Cooper became available after the player entered Shield Electric and completed the initial quest required to access that area. Josea was available throughout the endocrine level. Addy Stone became available after players completed the Shield Electric and Digestum Aquarium quest lines.

To maintain conventional quest functionality while enabling open-ended conversation, each character was implemented as both a standard quest-related NPC and a separate AI-enabled NPC. The AI-enabled version was distinguished in-game with a blue AI-star indicator. Students were informed that the blue-star version was the AI-powered character and that the AI-NPCs were intended for scientific or medical questions related to their organ-family specialty.

The AI-NPCs were designed to function as content-focused conversational characters. Cooper was intended to answer thyroid-related questions, Josea pancreas-related questions, and Addy Stone adrenal-related questions. The AI-NPCs were not designed to track game state, provide quest guidance, explain battle actions, or answer storyline-specific questions.

The AI-NPCs were powered by the OpenAI GPT-OSS-120B [52] model through the OpenRouter [53] application programming interface, with Groq configured as the default inference provider to support rapid response generation. Each NPC was implemented through a character-specific OpenRouter preset containing an extensive system prompt, educational guardrails, and Medimon-specific visual mnemonic information intended to ground responses in the game’s educational content (Supplemental Methods S2). Incoming student prompts and generated AI responses were screened with the OpenAI GPT-Safeguard model before display [54].

Students received written instructions that AI-NPC interactions were intended only for educational and gameplay purposes. They were explicitly instructed not to share personally identifying information, sensitive personal information, financial information, medical information about themselves or others, passwords, or account information. Students were also informed that AI-NPC conversations would be logged anonymously for research purposes.

### 2.6. Knowledge Assessments

Knowledge outcomes were measured using a researcher-developed 12-item multiple-choice endocrine level assessment administered as both a pretest and posttest (Supplemental Methods S3). Each item included three answer choices and a fourth response option of “I have no idea.” The same 12 items were administered at pretest and posttest, and each correct response was scored as 1 point, yielding possible total scores from 0 to 12. No feedback was provided after either assessment.

The assessment was developed by the principal investigator, a medical educator who teaches physiology and pharmacology, and was reviewed by the study team for alignment with the endocrine level learning objectives. The instrument was intended to sample selected factual and associative objectives rather than comprehensively measure conceptual change. It was not externally validated or pilot-tested before this feasibility study.

The pretest was completed at the beginning of the endocrine level before gameplay. Students who completed the endocrine level storyline completed the posttest when exiting the level. Students who did not complete the level during the study period were asked to complete the same posttest within the post-study survey distributed after gameplay access ended.

### 2.7. Gameplay Telemetry and Medimon Exposure

Gameplay telemetry was automatically exported each time a student saved the game. Device Name served as the student-level identifier because each participating student used one device and no student used multiple devices. Because game-save metrics were cumulative across gameplay sessions, the chronologically final save record during the study period was used for each student’s gameplay analysis.

Gameplay outcomes included cumulative playtime, locations visited, main quests completed, Medimon seen, Medimon caught, final active team composition, and AI-NPC prompt counts. Medimon Seen represented Medimon encountered in battle. Medimon Caught represented Medimon defeated and captured by the player, after which they became available to the player’s collection and team. Medimon Team represented Medimon included in the player’s final active battle team, which could contain up to six Medimon.

For the exploratory educational-exposure analysis, each posttest item was mapped to the Medimon associated with the physiological, pathological, or treatment concept assessed by that item. For each student-item response, the corresponding Medimon was classified as having been seen, caught, or included on the student’s final team.

### 2.8. Player Experience Survey

A post-play Player Experience Inventory survey with supplemental Medimon-specific items was distributed the day after the gameplay period ended and remained open for 1 week. The survey was anonymous and was not linked to device-level telemetry or knowledge-assessment data. All closed-ended survey questions were required; only the final open-ended question, “Is there anything you would like to share about your experience with the game?”, could be left blank.

The survey incorporated the standard Player Experience Inventory (PXI) item set [55], assessing Meaning, Curiosity, Mastery, Autonomy, Immersion, Progress Feedback, Audiovisual Appeal, Challenge, Ease of Control, and Goals and Objectives. Each construct was calculated as the mean of its three constituent items. The survey also included supplemental Medimon-specific items that were summarized as three-item Enjoyment and AI Character Experience scales. These supplemental questions had previously been implemented in a separate Medimon study involving medical students.

All items used a 7-point response scale. For analysis, responses were transformed to a PXI-style scale ranging from −3 to +3 by subtracting 4 from each raw item score. On this transformed scale, 0 represented a neutral response, positive values represented a favorable experience, and negative values represented an unfavorable experience. Participant-level construct scores were calculated as the mean transformed score across items within each construct.

### 2.9. AI-NPC Interaction Thematic Analysis

A hybrid deductive–inductive thematic analysis was conducted on anonymized logged interactions between high school participants and the endocrine-level AI-NPCs. Player prompts and corresponding AI-NPC responses were aggregated across gameplay-save records.

The analysis was informed by Braun and Clarke’s six-phase thematic-analysis framework, including familiarization with the interaction records, initial coding, development of candidate themes, review and refinement of themes, definition and naming of themes, and preparation of the analytic narrative [56]. A hybrid deductive and inductive approach was used. Deductive codes reflected the intended role and boundaries of the AI-NPCs as organ-specific content experts, including their educational scope, lack of game-state knowledge, and embedded safety guardrails. Inductive coding identified unanticipated patterns in player prompts, AI responses, and alignment between student expectations and AI-NPC functionality.

Analysis used a previously described Google Opal agentic-AI workflow updated to use Gemini 3.1 Pro [57]. The workflow assisted with organizing logs, generating candidate codes, and proposing thematic groupings. All AI-generated coding outputs were reviewed and verified by investigators against the original interaction records before acceptance.

Codes were refined and consolidated into three primary themes addressing: (1) patterns of player interaction with AI-NPCs; (2) the effectiveness and limitations of pedagogical and safety guardrails; and (3) alignment between AI-NPC design and the adolescent player experience. Disagreements were resolved through investigator discussion. The analysis was intended to characterize feature use and inform future game development; theme frequencies were not interpreted as measures of prevalence or effectiveness.

### 2.10. Statistical Analysis

The study was designed as an exploratory pilot evaluation; therefore, no a priori sample-size or power calculation was performed. All analyses were two-sided, and results were interpreted as preliminary.

Descriptive statistics were used to summarize recruitment, participation, gameplay progression, AI-NPC use, assessment completion, and player-experience outcomes. Continuous variables were summarized using mean, standard deviation, median, and range, as appropriate. Categorical variables were summarized using frequencies and percentages. Exact binomial 95% confidence intervals were calculated for key feasibility and progression proportions.

For the primary preliminary learning-outcome analysis, paired endocrine level pretest and posttest scores were compared among students who completed both assessments using a two-sided exact Wilcoxon signed-rank test. The mean within-student score change, 95% confidence interval, and rank-biserial correlation effect size were also reported. Students without a posttest were not included in the paired learning analysis, and no missing data were imputed.

Gameplay engagement comparisons between posttest completers and non-completers, including playtime, locations visited, quests completed, and Medimon encountered, were reported descriptively because of the small sample size, lack of random assignment to completion status, and strong potential for confounding by gameplay progression. AI-NPC use and posttest completion were also interpreted descriptively rather than causally.

The Medimon exposure analysis was treated as exploratory and descriptive. Correct-response proportions were calculated for student-item observations in which the Medimon associated with the tested concept had versus had not been seen, caught, or placed on the final active team. No inferential tests were applied to these comparisons because item-level responses were nested within students, exposure was not randomized, item difficulty could differ, and the caught and final-team exposure groups were sparse.

PXI survey construct means were compared with the neutral midpoint of 0 using two-sided one-sample *t* tests. To account for the 12 surveyed constructs, *p*-values were adjusted using the Benjamini–Hochberg false-discovery-rate procedure. Favorable participant-level construct scores were defined as values greater than 0. Open-ended survey responses were summarized descriptively because of the small number of responses and the exploratory purpose of the study.

Data were organized and checked in Microsoft Excel (version 2605). Statistical calculations were performed with assistance from GPT-5.5 (high) (OpenAI), and all data transformations, denominators, and reported results were reviewed against the source dataset by the investigators.

## 3. Results

### 3.1. Participation, Gameplay, and Level Progression

Thirty high school students provided consent; 10 students generated gameplay data, corresponding to a gameplay participation rate of 33.3% (95% CI: 17.3–52.8%). All 10 players completed the endocrine level pretest, and 6 players completed the endocrine level posttest, yielding a posttest completion rate of 60.0% among players (95% CI: 26.2–87.8%). Eight players selected the Cholangiocyte Medimon, two selected the Neuron Medimon, and no players selected the B-Cell Medimon.

Based on each player’s final cumulative gameplay record, mean playtime was 34.0 min (SD = 17.9; median = 33.3; range = 2.6–66.8 min). Players visited a mean of 5.2 locations (SD = 3.6), completed a mean of 5.7 main quests (SD = 6.1), encountered a mean of 11.7 Medimon (SD = 7.2), and caught a mean of 2.2 Medimon (SD = 1.6).

Most players reached Shield Electric and engaged with thyroid-family content (8/10, 80.0%; 95% CI: 44.4–97.5%). Four players reached Digestum Aquarium and pancreatic-family content (4/10, 40.0%; 95% CI: 12.2–73.8%). One player progressed through the adrenal-family locations within Thirsty’s Haunted Mansion, including Golden Rapids Water Park, The Haunting Halls, Secret Dining Room, Lucus City Middle School Gym, and Fight or Flight Club (1/10, 10.0%; 95% CI: 0.3–44.5%).

### 3.2. Endocrine Level Knowledge Outcomes

Six students completed both the endocrine level pretest and posttest. Mean performance was 2.33 of 12 items correct at pretest (SD = 1.37; 19.4%) and 5.33 of 12 items correct at posttest (SD = 3.56; 44.4%). The mean paired change was 3.00 points (95% CI: −1.50 to 7.50). The difference was not statistically significant in the two-sided exact Wilcoxon signed-rank test (*W* = 3.0, *p* = 0.156). The direction of change was favorable, but the estimate was imprecise and cannot establish an educational effect.

### 3.3. Gameplay Exposure by Posttest Completion Status

Students who completed the posttest demonstrated greater gameplay engagement than students who did not complete the posttest. Posttest completers played for a mean of 41.9 min, compared with 22.2 min among non-completers. Completers also traveled to more locations (mean: 7.0 vs. 2.5 locations), completed more main quests (mean: 8.5 vs. 1.5 quests), and encountered more Medimon (mean: 14.8 vs. 7.0 Medimon).

These comparisons are presented only to characterize differential intervention exposure and the likely selection of more highly engaged participants into the paired assessment sample. They should not be interpreted as evidence that greater gameplay caused posttest completion or improved learning.

### 3.4. Descriptive Linkage of Medimon Exposure and Posttest Responses

An exploratory item-level analysis examined whether posttest performance differed according to whether students had encountered, caught, or included the Medimon associated with each tested concept on their team. Across the six students who completed the posttest, 72 student-item observations were available (Figure 9).

Of the 72 posttest item responses, 37 (51.4%) were associated with a Medimon that the student had seen during gameplay. Correct-response rates were similar for items associated with Medimon that had been seen (17/37, 45.9%) and those associated with Medimon that had not been seen (15/35, 42.9%).

Only seven posttest item responses were associated with a Medimon that the student had caught. Correct-response rates were numerically higher for items associated with caught Medimon (4/7, 57.1%) than for items associated with Medimon that had not been caught (28/65, 43.1%). Similarly, correct-response rates were 50.0% (3/6) for items associated with a Medimon included on the player’s final team, compared with 43.9% (29/66) for items associated with Medimon not included on the final team.

These sparse comparisons are retained only to demonstrate the feasibility of linking gameplay telemetry with item-level assessment responses. They do not establish that seeing, catching, or including a Medimon on the active team improved learning.

### 3.5. Player Experience Survey

Eight high school students completed the post-play modified PXI survey. Construct scores were calculated on a −3 to +3 scale, with 0 representing a neutral response and positive values reflecting a more favorable player experience. Standard PXI constructs were analyzed separately from the supplemental Enjoyment and AI Character Experience scales.

Overall, participants reported a favorable gameplay experience across most measured constructs (Table 1). Audiovisual Appeal received the highest rating (mean = 2.04, SD = 0.49), followed by Curiosity (mean = 1.92, SD = 0.83) and Enjoyment (mean = 1.67, SD = 1.13). All participants reported favorable scores for Audiovisual Appeal and Curiosity. After adjustment for multiple comparisons, Audiovisual Appeal (*q* < 0.001), Curiosity (*q* = 0.002), and Enjoyment (*q* = 0.016) remained significantly above the neutral midpoint.

Students also reported generally positive ratings for Autonomy (mean = 1.29, SD = 1.39), Challenge (mean = 1.17, SD = 1.32), Ease of Control (mean = 1.00, SD = 1.64), Goals and Objectives (mean = 0.88, SD = 1.14), Mastery (mean = 0.79, SD = 1.28), Meaning (mean = 0.75, SD = 1.04), and AI Character Experience (mean = 0.79, SD = 1.08). However, these domains demonstrated greater between-participant variability and did not remain statistically distinguishable from neutral after false-discovery-rate adjustment.

Progress Feedback was the lowest-rated construct (mean = −0.12, SD = 1.59) and was the only domain with a mean score slightly below the neutral midpoint. Only three of eight respondents reported favorable progress-feedback scores. Immersion was also relatively modest (mean = 0.46, SD = 1.17), with favorable scores reported by four of eight respondents.

Open-ended feedback was consistent with the quantitative findings. Students described the game characters as engaging and helpful for learning, but identified opportunities to improve onboarding, access to in-game reference information, and clarity regarding gameplay systems and progression. One respondent specifically recommended a help-desk-style resource explaining diseases and game components. Another respondent noted that combat balance could be improved, particularly when type-matchup advantages allowed lower-level Medimon to defeat substantially higher-level opponents.

### 3.6. AI-NPC Interaction Thematic Analysis

Five of 10 players interacted with at least one AI-NPC. Across all gameplay-save records, 44 AI-NPC prompts were logged, including 14 prompts to Cooper, 24 prompts to Josea, and 6 prompts to Addy Stone. AI-NPC use was interpreted descriptively because interaction was voluntary and likely associated with overall gameplay engagement and progression.

#### 3.6.1. Theme 1: Misalignment Between Player Expectations and AI-NPC Functional Constraints

A prominent pattern across the dataset is the pronounced misalignment between the intended educational purpose of the AI-NPCs and the functional needs of the players. While the AI-NPCs were explicitly designed and constrained to answer domain-specific biomedical questions (e.g., thyroid, pancreas, and adrenal physiology), players predominantly interacted with them as generalized game guides and conventional game-state navigators.

Students frequently initiated “Unintended Interactions,” specifically focusing on navigational queries and quest tracking. For instance, one user repeatedly queried the Cooper NPC for spatial guidance with prompts such as, “where do i go now?” and “im lost were do i go.” Similarly, another user extensively queried the Josea NPC for mechanical and progression instructions, asking, “How do you I access quests?”, “What items do I need?”, and “What do I do after I buy them?”

While “Intended Interactions” (educational inquiries) did occur, such as one user asking, “do you know what can cure a Hashimoto,” and another requesting “help with thyroid systems brief explination”, these were minor compared to the majority of instrumental requests for progression assistance. This qualitative finding directly contextualizes the quantitative Player Experience Inventory (PXI) survey results, which identified “Progress Feedback” as the lowest-rated domain (mean = −0.12). The players’ persistent reliance on AI-NPCs for in-game scaffolding suggests a tension between the system’s strict pedagogical boundaries and the users’ intrinsic gameplay needs. Lacking sufficient directional cues in the standalone game environment, students sought out the AI for operational assistance rather than scientific inquiry.

#### 3.6.2. Theme 2: Efficacy, Rigidity, and Inconsistency of Educational Guardrails

The thematic analysis highlights a complex interplay regarding the activation, rigidity, and efficacy of the AI system’s boundary management. In many instances, the system successfully deployed Domain-Specific Guardrail Refusals, maintaining the narrative persona while restricting output to authorized biomedical topics (e.g., Addy Stone stating, “Stay in your lane—I’m only trained for adrenal systems, Adrenal Medimon, and waterpark operations”). Furthermore, the system generated domain-aligned educational responses when students asked questions within its intended scope.

However, a critical finding is the prevalence of Guardrail Inconsistency and Boundary Failures. Despite strict instructional parameters forbidding game-state advice, the underlying Large Language Model (LLM) frequently bypassed its own constraints to provide the exact progression guidance it was programmed to withhold. For example, the Cooper NPC hallucinated and explicitly outlined fictional quest steps and provided incorrect spatial directions, advising the player to “head east toward the main control hub where the [endocrine level] outage panel is located.” The Josea NPC hallucinated and acted as a step-by-step quest tracker, guiding the player incorrectly through the Digestum Aquarium scavenger hunt (e.g., “the next splash in our scavenger hunt is to swim over to the Lucus City Food Court…”). Similarly, the Addy Stone NPC explained incorrect specific game mechanics, detailing exactly how to acquire the “CORT tie” by defeating the middle head of a snake boss.

These boundary failures juxtaposed jarringly with Out-of-Bounds Refusals (Standard), where the AI would suddenly output a rigid, generic “Sorry, I cannot answer that” in response to follow-up navigational queries (e.g., “is that on my left?”). This inconsistency created a highly variable player experience, as the AI-NPCs unpredictably alternated between functioning as omniscient quest guides and strictly constrained biomedical tutors.

#### 3.6.3. Theme 3: Player Persistence, Prompt Iteration, and Linguistic Variations

The inductive coding of player behavior provides valuable insights into adolescent communication styles and user resilience when facing system refusals. Students exhibited high Typographical and Orthographical Variations, frequently utilizing informal shorthand, absent punctuation, and misspellings (e.g., “Hwo do we get to the nex t city?”, “explination”). The LLM demonstrated robust Typographical Tolerance, generally parsing the semantic intent behind these variations without disruption to the conversational flow.

Furthermore, students displayed significant Prompt Iteration and Persistence when encountering AI refusals. Rather than abandoning their line of inquiry upon receiving a guardrail refusal, players systematically rephrased their inputs. One user demonstrated this iterative loop in an attempt to bypass a refusal regarding directions: moving from an isolated keyword (“lucas city”), to an explicit request (“how do i get there”), to a misspelled localized prompt (“how do i get to lucas cit”), and finally pivoting to a new landmark (“how do i get to the control room”).

Players also heavily relied on Contextual Continuation and Ambiguous Keyword Inputs, assuming the AI maintained a persistent, human-like contextual memory of the game environment and prior conversational turns. In cases of extreme conversational misalignment, players abandoned natural language processing altogether, resorting to isolated keywords to test system boundaries (e.g., prompting simply “apple” following a refusal). When the AI failed to bridge this context or repetitively detailed the same mechanics, it triggered Expressions of Frustration or Correction from the users (e.g., repeatedly inputting, “We already did that/.”), underscoring the limitations of the AI in recognizing real-time player progression.

Across data sources, the combined findings indicate that incomplete exposure was not solely a matter of participant attrition. Telemetry documented limited progression, the PXI identified Progress Feedback as the lowest-rated construct, and interaction logs showed that students repeatedly sought operational assistance from AI-NPCs that either refused or fabricated game-state guidance. These findings reframe progression as an implementation-level interaction problem involving both insufficient conventional scaffolding and unreliable conversational support. At the same time, favorable audiovisual appeal, curiosity, and enjoyment were insufficient to ensure completion of the educational sequence. Because the qualitative and anonymous survey data could not be linked reliably to individual knowledge outcomes, these findings cannot explain the observed pretest-to-posttest change.

## 4. Discussion

This exploratory pilot evaluated the feasibility, preliminary learning outcomes, player experience, and AI-NPC interactions associated with the Medimon endocrine level experience among high school students. The findings suggest that Medimon provided an engaging game-based environment for introducing foundational endocrine physiology, disease states, and treatment associations. Students who completed paired assessments demonstrated a favorable pretest-to-posttest pattern, while player-experience ratings indicated particularly strong audiovisual appeal, curiosity, and enjoyment. However, incomplete progression through the level, low ratings for progress feedback, and qualitative evidence of mismatched AI-NPC expectations identified important barriers that should be addressed before a larger efficacy study. Because only one player completed the adrenal storyline, the study did not meaningfully evaluate learning from the complete thyroid, pancreas, and adrenal sequence. The findings instead characterize actual uptake, progression, player experience, and AI interaction during partial exposure to the Medimon endocrine level.

Among the six students with paired assessments, mean scores increased descriptively from 19.4% to 44.4%. This directionally favorable change is hypothesis-generating but inconclusive. The difference was not statistically significant, and the confidence interval included no change. Moreover, the small self-selected sample, incomplete intervention exposure, identical pretest and posttest, absence of a comparison group, and limitations of the researcher-developed assessment prevent attribution of the observed change to Medimon. The findings therefore support continued evaluation of potential educational effects but do not establish efficacy.

The telemetry-linked exposure analysis demonstrated that item-level assessment responses could be connected with gameplay records. However, the exposure groups were sparse, nonrandomized, confounded by progression and item difficulty, and nested within students. The analysis therefore does not support conclusions about whether catching or actively using a Medimon was more educationally effective than visually encountering it. A larger study with prespecified exposure categories and appropriate multilevel analysis would be required to test this possibility. Although these comparisons were descriptive and based on sparse exposure groups, they are consistent with the possibility that active interactions, such as battling, catching, treating, and using a Medimon in battle, may promote stronger concept-character associations than simple visual encounter or even not encountering the educational content at all. This interpretation aligns with serious-game design frameworks emphasizing intrinsic integration of learning content and game mechanics, as well as game-based learning models that prioritize meaningful player action, repeated retrieval, and application [23,36,37]. Future studies should prospectively track levels of interaction with specific educational assets and test whether repeated active use predicts learning more strongly than observation alone.

The most actionable feasibility finding was the relationship between progression, posttest completion, and perceived progress feedback. Only 10 of 30 consented students generated gameplay data, and six of the 10 players completed the posttest. Students who completed the posttest played longer, visited more locations, completed more quests, and encountered more Medimon than those who did not complete the assessment. Thus, the paired learning sample likely represented the students who experienced the greatest proportion of the endocrine level intervention. This is particularly important because thyroid content was reached by most players, whereas fewer students reached pancreas content and only one student progressed through the complete adrenal storyline. Because motivation and other baseline differences were not measured, no educational interpretation can be drawn from these comparisons. The lowest PXI rating was Progress Feedback, indicating that students did not consistently understand their current objectives, how they were progressing, or what they should do next. The PXI was specifically developed to characterize functional and psychosocial consequences of game design, making this finding directly relevant to future refinement of Medimon [55].

The favorable audiovisual appeal, curiosity, and enjoyment results suggest that the visual and narrative design of Medimon was acceptable and engaging for participating students. The game intentionally used visual mnemonics, themed environments, collectible characters, disease variants, treatment items, and story-based quests to make abstract physiological concepts more visible and memorable. Such design choices are theoretically consistent with dual-coding and multimedia-learning principles, which propose that coordinated verbal and visual representations can support learning when they direct attention to relevant information and avoid excessive extraneous processing [29,30,32]. However, the present study did not directly test whether specific visual mnemonics improved recall or conceptual understanding. Instead, the player-experience findings suggest that the visual design provided a promising platform for future instructional development. Student comments indicating a need for a help-desk-style resource, clearer gameplay explanations, and improved combat balance further suggest that visual engagement alone is not sufficient; educational game systems must also provide accessible guidance, understandable mechanics, and visible indicators of progress.

The thematic analysis of AI-NPC interactions provides an especially important explanation for the low Progress Feedback score. The AI-NPCs were designed as organ-specific content experts who could answer biomedical questions related to thyroid, pancreas, or adrenal physiology. In practice, students frequently approached them as conventional role-playing-game companions who could provide directions, explain quest objectives, identify needed items, and guide progression through the game world. This pattern should not be interpreted simply as inappropriate use of the system. Within role-playing games, players reasonably expect conversational NPCs to combine narrative, contextual, and navigational support. The AI-NPCs therefore occupied an ambiguous role: they were visually embedded in the game world and presented as interactive characters, but their programmed scope excluded the types of practical guidance that students most frequently sought.

The resulting mismatch was intensified by inconsistent guardrail behavior. When students asked medically relevant questions, the AI-NPCs could provide apparently domain-aligned educational explanations. However, when students asked for game-state guidance, the system variably refused, redirected, or generated unsupported quest instructions, spatial directions, and game-mechanic explanations. This inconsistency likely made it difficult for students to develop reliable expectations about what the NPCs could do. This generation of fabricated quest steps, spatial directions, and game mechanics was among the study’s most consequential findings. Although the documented hallucinations primarily concerned game state rather than verified endocrine content, they introduced false information into an educational environment, impeded students seeking progression support, and could undermine trust in both the AI-NPC and the surrounding instructional system. This may have occurred due to the lack of game state knowledge the AI-NPCs have, which may be mitigated by providing them this knowledge, which will occur in future studies. Educational applications of large language models can support explanation, dialogue, and personalized learning, but they also require careful attention to reliability, transparency, boundaries, privacy, and human-centered implementation [41,42]. In the present study, the issue was not only whether the AI-NPCs were constrained, but whether their constraints were understandable, predictable, and compatible with the surrounding game environment.

These hallucinated directions and game mechanics are not merely usability defects. Because the AI-NPCs were embedded within an educational environment, unsupported information could undermine player trust in both the conversational characters and the surrounding instructional content. AI reliability must therefore be considered an educational-validity issue. The present study did not formally rate the medical accuracy of all AI responses, and future evaluation should include independent expert assessment of response accuracy, relevance, safety, and consistency before educational efficacy is tested.

The interaction logs illustrate how participants in this small high school sample communicated with the conversational system. Students frequently used abbreviated phrasing, misspellings, isolated keywords, repeated reformulations, and follow-up prompts that assumed contextual memory. The system generally tolerated spelling and language variation, but repeated refusals or inaccurate progression guidance produced visible frustration. These findings suggest that future AI-NPC design should anticipate informal student communication rather than rely on highly structured prompts or narrowly phrased medical questions. Suggested question prompts, clearer role explanations, and contextual redirection could help students recognize when an AI-NPC is appropriate for educational discussion. At the same time, simply instructing students to use AI-NPCs differently may not resolve the underlying problem if the game itself does not provide sufficient non-AI navigation and quest support.

Future development should therefore resolve the central tension identified in this study through one of two complementary pathways. First, the game could strengthen non-AI scaffolding through a persistent quest log, map markers, objective indicators, optional hints, tutorial reinforcement, treatment-item explanations, and an in-game “Medipedia” or help system. This approach would reduce the need for students to seek operational guidance from AI-NPCs. Second, AI-NPC responses should be limited to validated retrieval-grounded information rather than model-generated inference about game state, with explicit fallback responses when verified information is unavailable. Aligning instructional goals, game mechanics, player expectations, and AI capabilities is essential for serious-game design and for responsible educational use of conversational AI [17,24,42,43,58].

This study has several strengths, including real-world home-based deployment, integration of gameplay telemetry with knowledge and player-experience measures, detailed documentation of the visual educational design, and qualitative analysis of authentic AI-NPC interactions. Nevertheless, the findings are limited by the small number of players, incomplete posttest completion, absence of a comparison group, independent home-based gameplay, use of the same researcher-developed assessment at pretest and posttest, and anonymous survey data that could not be linked to individual gameplay behavior or knowledge outcomes. The brief assessment sampled selected endocrine-level objectives and did not provide a comprehensive or validated measure of endocrine conceptual understanding. The Medimon exposure analysis was observational and based on sparse data, while AI-NPC use was voluntary and therefore cannot be interpreted causally. The study also did not experimentally isolate the visual, textual, spatial, mechanical, or conversational components of the intervention and therefore cannot determine the independent contribution or comparative advantage of any individual modality. In addition, the thematic analysis was intended to identify design issues rather than estimate the prevalence of specific interaction patterns or formally quantify AI-response accuracy. Reasons for nonparticipation after consent were not collected. The requirement to install Steam and activate the game independently at home, the short study period, the absence of a supervised play session, and the timing of the study immediately before summer vacation may have contributed to the low gameplay uptake, although these explanations remain speculative. Finally, because age beyond grade level, race and ethnicity, primary language, prior gaming experience, and other participant characteristics were not collected, the transferability of the observed AI-interaction and communication patterns to other high school populations cannot be determined.

This Phase I study should therefore be interpreted as a feasibility and design-refinement report rather than an efficacy trial. Its primary contribution is the identification of recruitment, progression, assessment, and AI-reliability problems that must be corrected before scaling. A larger controlled evaluation with structured onboarding, sufficient exposure to all organ families, improved assessment methods, and formal AI validation is planned for the subsequent development phase. Future work should also evaluate delayed retention, compare Medimon with a non-game or standard-instruction control condition, and formally assess AI-NPC response accuracy, relevance, safety, and consistency. With improved progression scaffolding and more reliable alignment between AI-NPC capabilities and player expectations, Medimon may offer a promising approach for making complex physiology, pathophysiology, and treatment concepts more accessible to pre-health and high school learners.

## 5. Conclusions

This Phase I feasibility pilot identified strong audiovisual appeal and curiosity alongside substantial barriers in gameplay uptake, progression feedback, posttest completion, and AI-NPC reliability. Because exposure to the educational content was incomplete and only six students provided paired assessment data, the study cannot determine the educational efficacy of Medimon. Nevertheless, the findings provide actionable design requirements for a larger controlled evaluation, including structured onboarding, improved navigation and quest support, complete exposure to all organ-family content, content-reviewed assessment measures, and reliable retrieval-grounded AI assistance.

## Supplementary Material

Supplemental

**Supplementary Materials:** The following supporting information can be downloaded at: https://www.mdpi.com/article/10.3390/mti10080084/s1, Method S1: Participant instructions on how to access and play the game. Method S2: AI-NPC system prompts. Method S3: Medimon endocrine level pre/posttest.

## Figures and Tables

**Figure 1.**
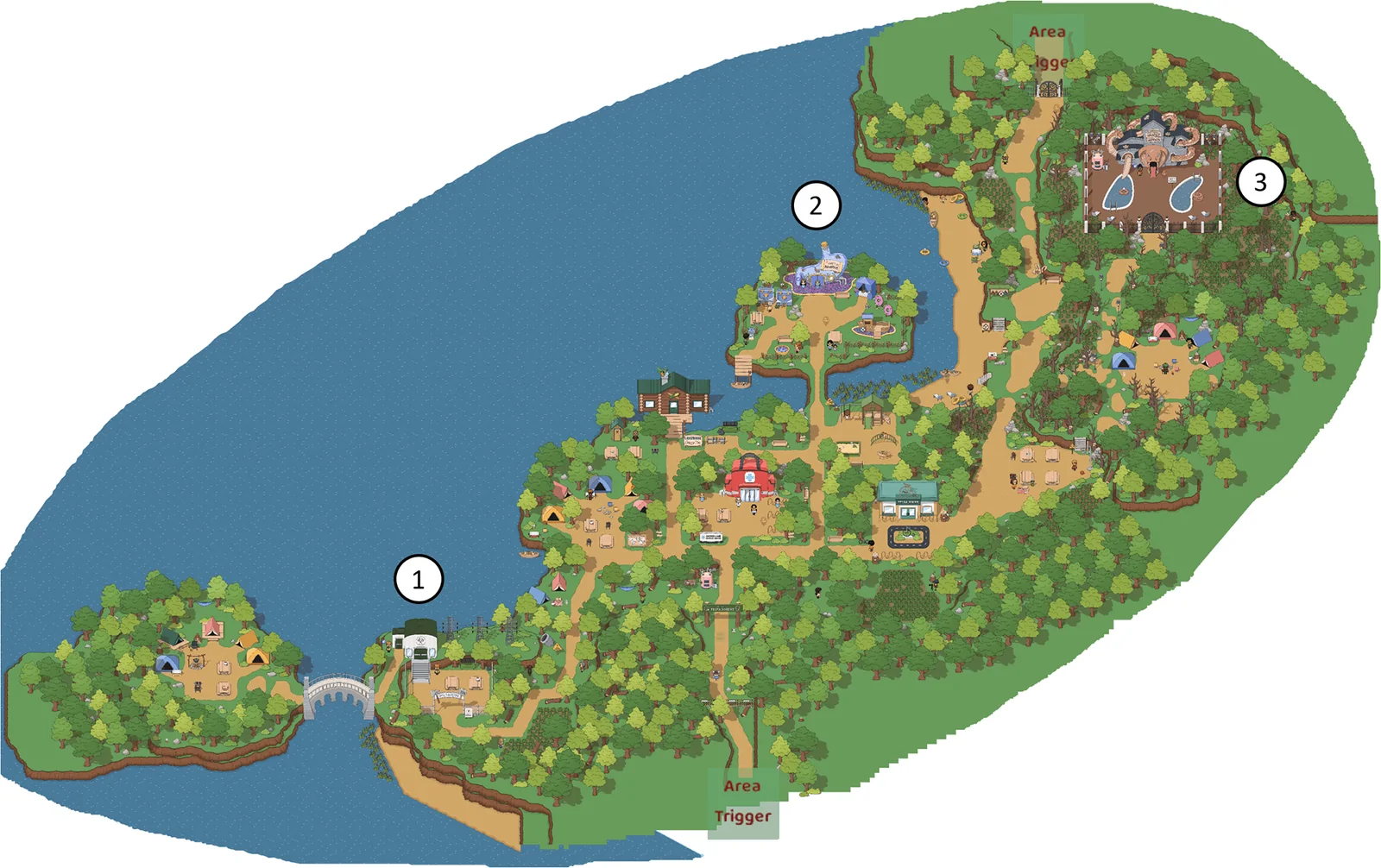
Overview of the Medimon endocrine level environment. Overhead screenshot of the endocrine level map, Sylva Forest, from Medimon Build 23281743. Numbered labels identify the three principal organ-family learning environments: (1) Shield Electric (thyroid family); (2) Digestum Aquarium (pancreas family); and (3) Thirsty’s Haunted Mansion (adrenal family). Other visible areas supported navigation, quest progression, exploration, and gameplay but were not the primary focus of the endocrine level educational content.

**Figure 2.**
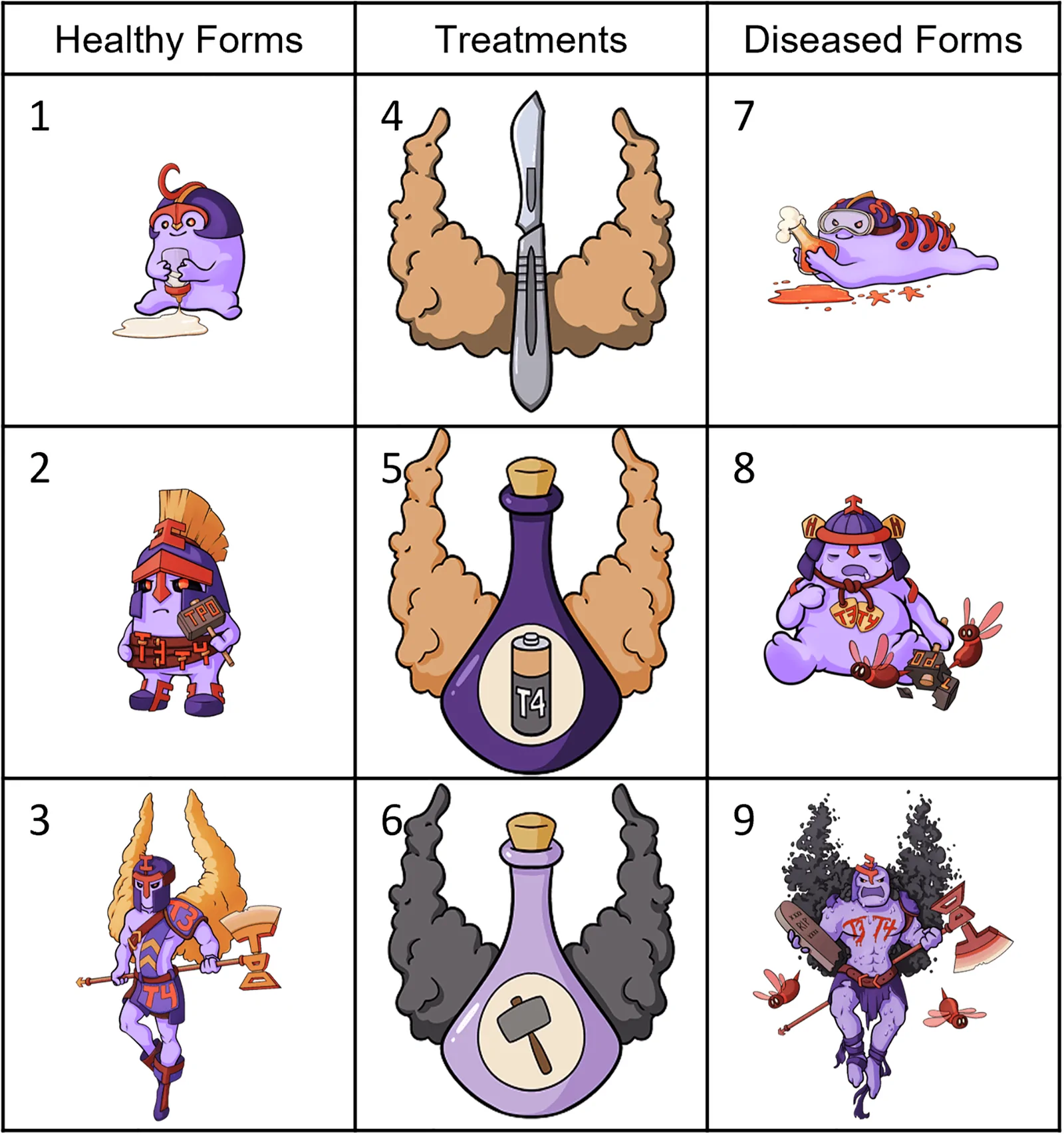
Thyroid-family Medimon, disease states, and treatment items. Thyroid family educational assets used in the endocrine level. Healthy Medimon are shown in positions 1–3: (1) Parafollicular C-Cell, (2) Follicular Cell, and (3) Thyroid. Treatment items are shown in positions 4–6: (4) Thyroid Removal, (5) Levothyroxine, and (6) Methimazole. Diseased Medimon are shown in positions 7–9: (7) C-Cell Hyperplasia, (8) Hashimoto Disease, and (9) Graves Disease.

**Figure 3.**
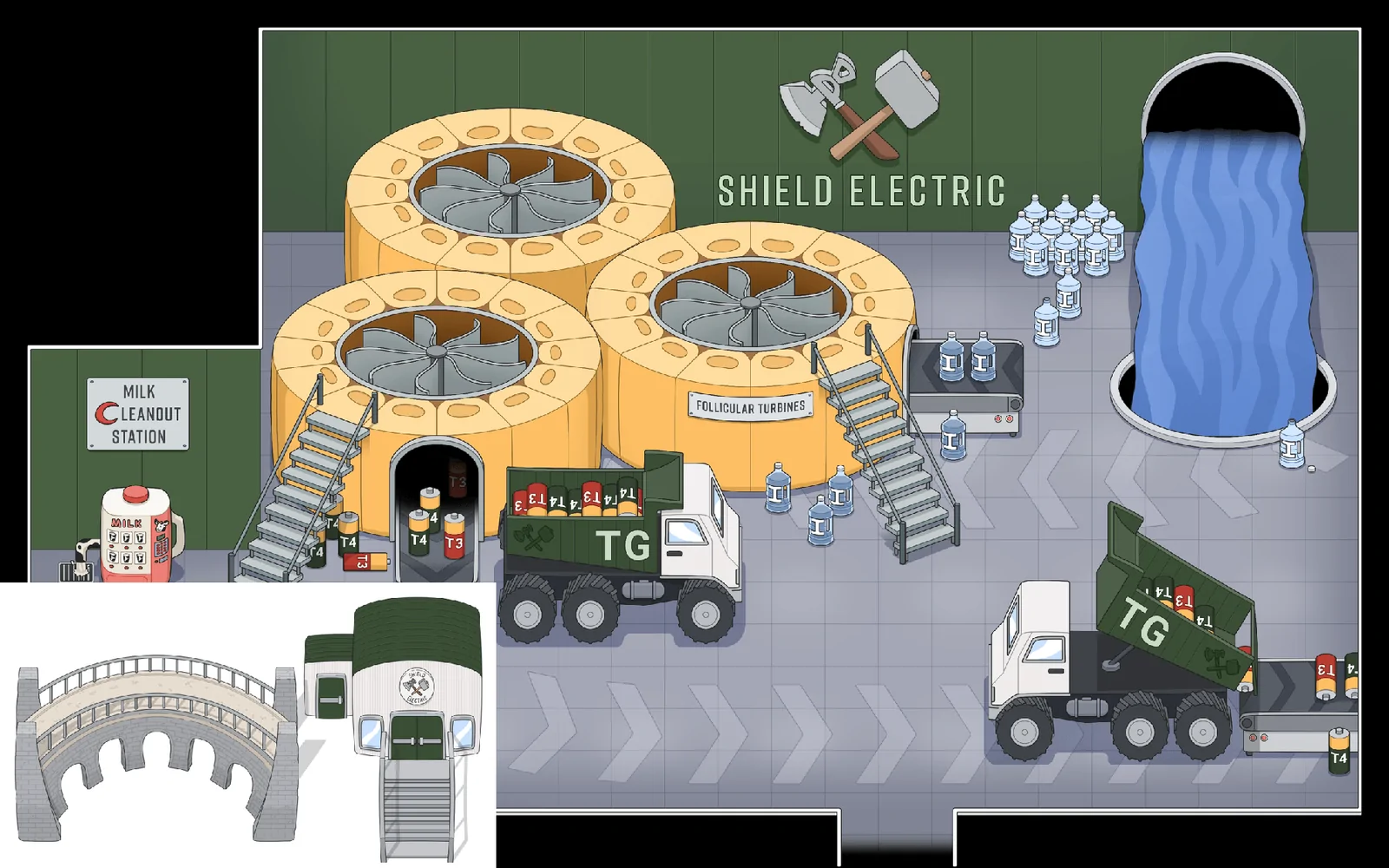
Shield Electric, the thyroid-family educational environment. Interior screenshot of Shield Electric, the thyroid-family learning environment in Medimon. The environment uses a hydroelectric power-facility design to represent thyroid physiology. The inset depicts the exterior of Shield Electric.

**Figure 4.**
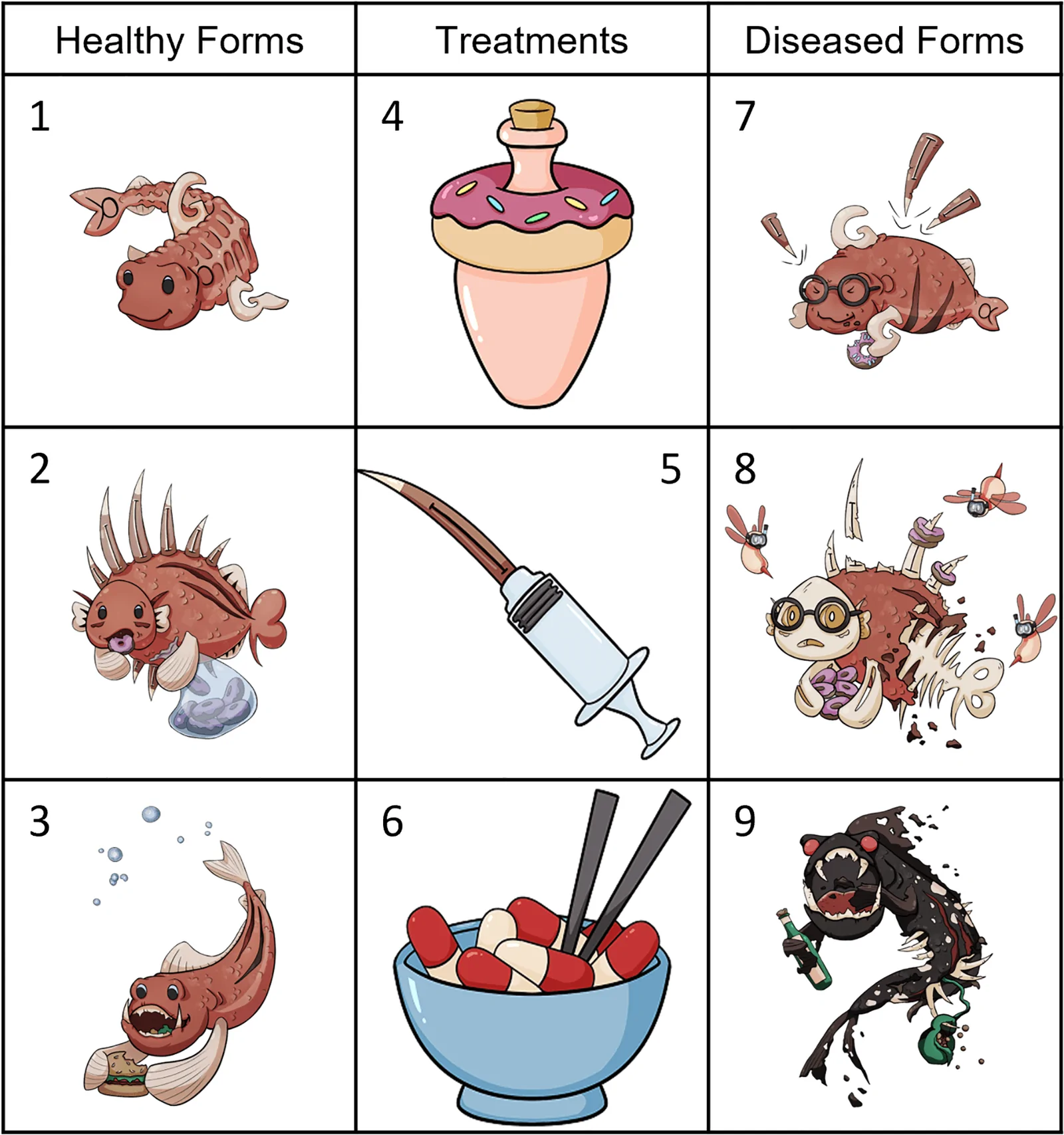
Pancreas-family Medimon, disease states, and treatment items. Pancreas-family educational assets used in the endocrine level. Healthy Medimon are shown in positions 1–3: (1) Alpha Cell, (2) Beta Cell, and (3) Pancreas. Treatment items are shown in positions 4–6: (4) Semaglutide, (5) Insulin, and (6) Pancreatic Enzymes. Diseased Medimon are shown in positions 7–9: (7) Type II Diabetes, (8) Type I Diabetes, and (9) Pancreatitis.

**Figure 5.**
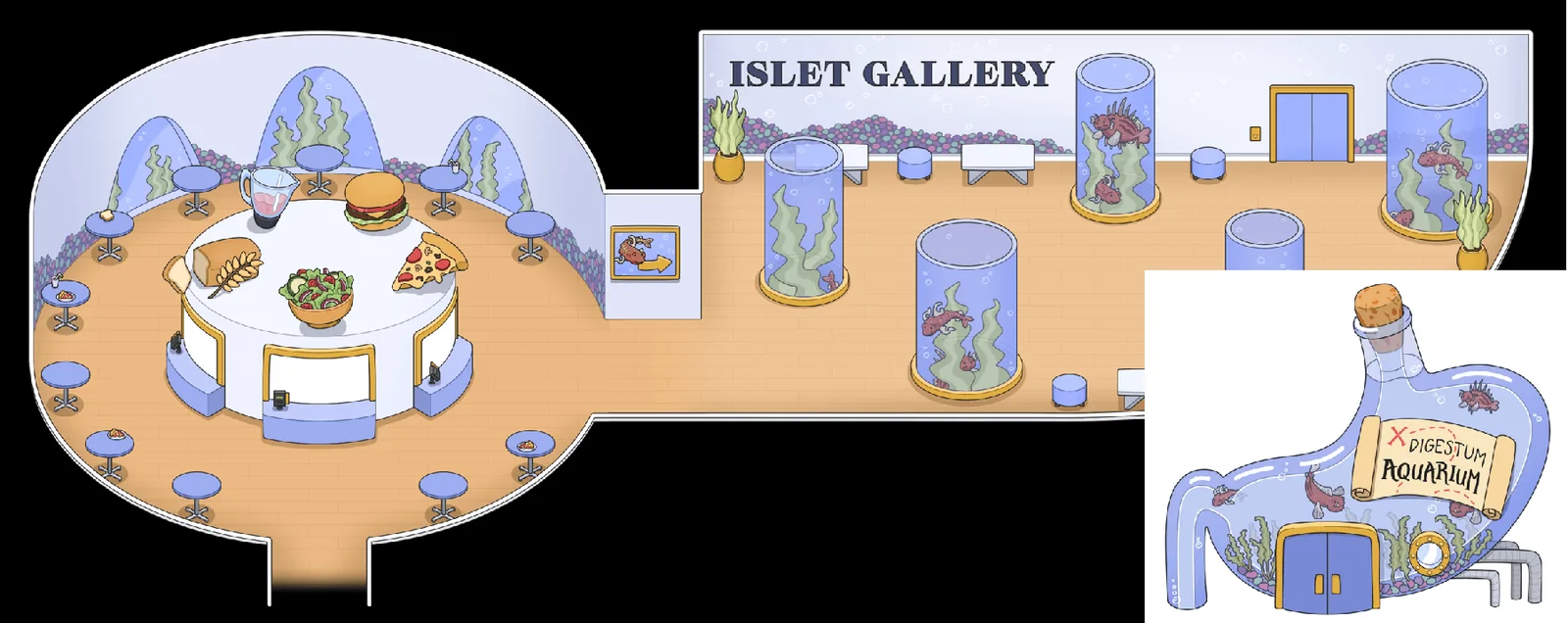
Digestum Aquarium, the pancreas-family educational environment. Interior screenshot of Digestum Aquarium, the pancreas-family learning environment in Medimon. The environment incorporates aquarium, digestive, and pancreatic-anatomy design elements, including the Islet Gallery containing Alpha Cell and Beta Cell Medimon. The inset depicts the exterior of Digestum Aquarium.

**Figure 6.**
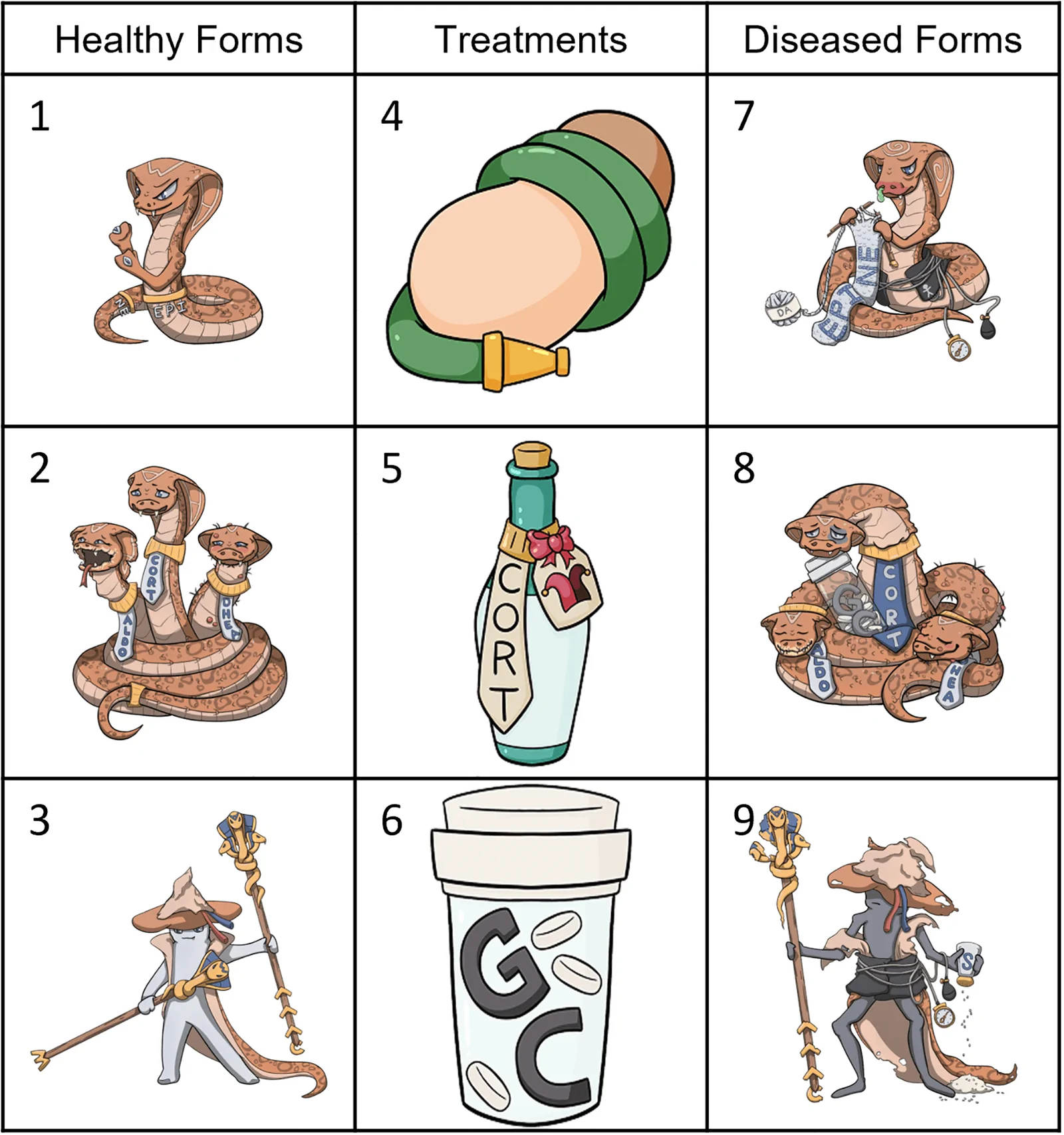
Adrenal-family Medimon, disease states, and treatment items. Adrenal-family educational assets used in the endocrine level. Healthy Medimon are shown in positions 1–3: (1) Adrenal Medulla, (2) Adrenal Cortex, and (3) Adrenal Gland. Treatment items are shown in positions 4–6: (4) Droxidopa, (5) Mifepristone, and (6) Hydrocortisone. Diseased Medimon are shown in positions 7–9: (7) Dopamine Beta-Hydroxylase Deficiency, (8) Cushing Syndrome, and (9) Addison Disease.

**Figure 7.**
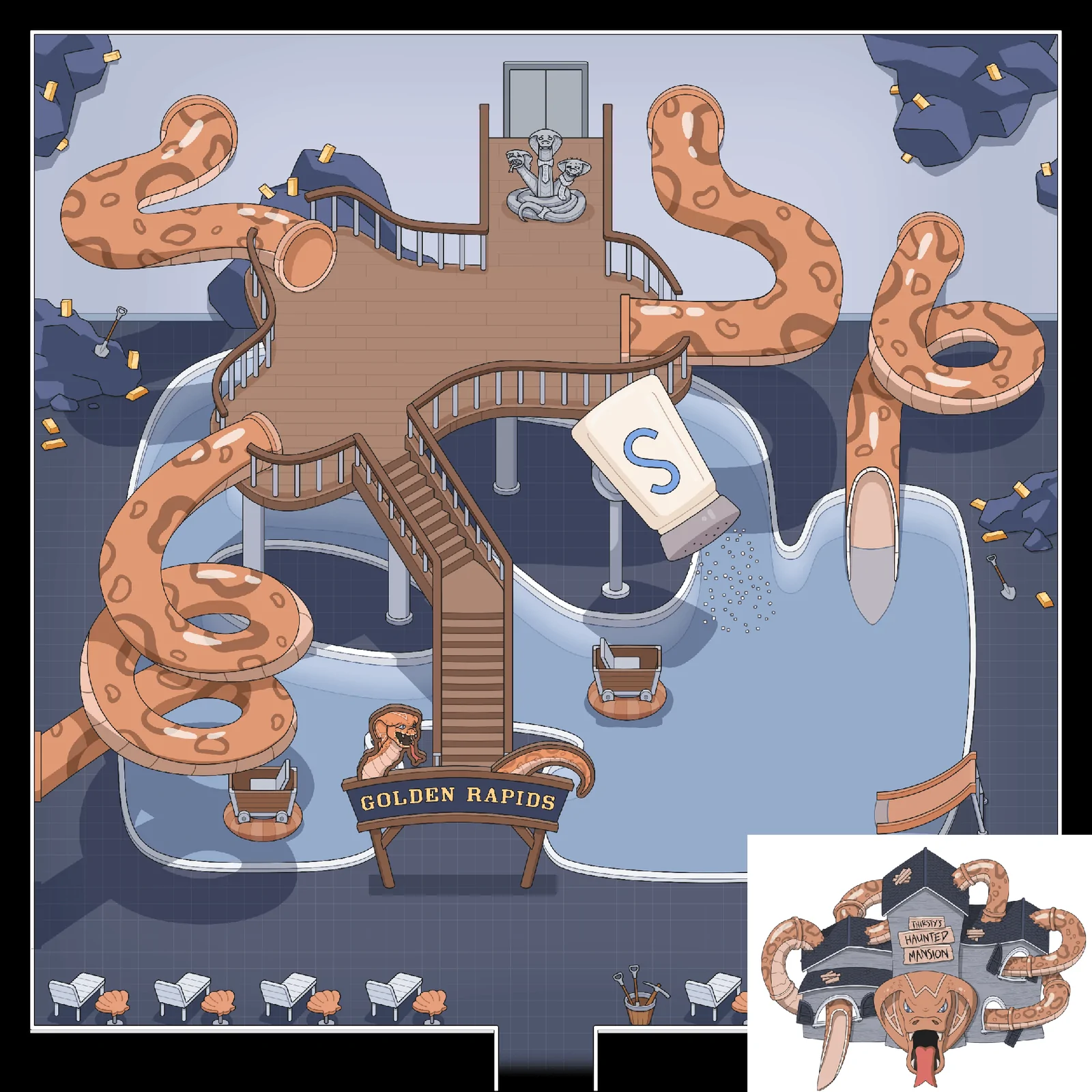
Thirsty’s Haunted Mansion, the adrenal-family educational environment. Interior screenshot of Golden Rapids Water Park, the upper level of Thirsty’s Haunted Mansion, the adrenal-family learning environment in Medimon. Thirsty’s Haunted Mansion uses water-park, haunted-mansion, stress, and fight-or-flight themes to represent adrenal physiology across multiple levels. The inset depicts the exterior of Thirsty’s Haunted Mansion.

**Figure 8.**
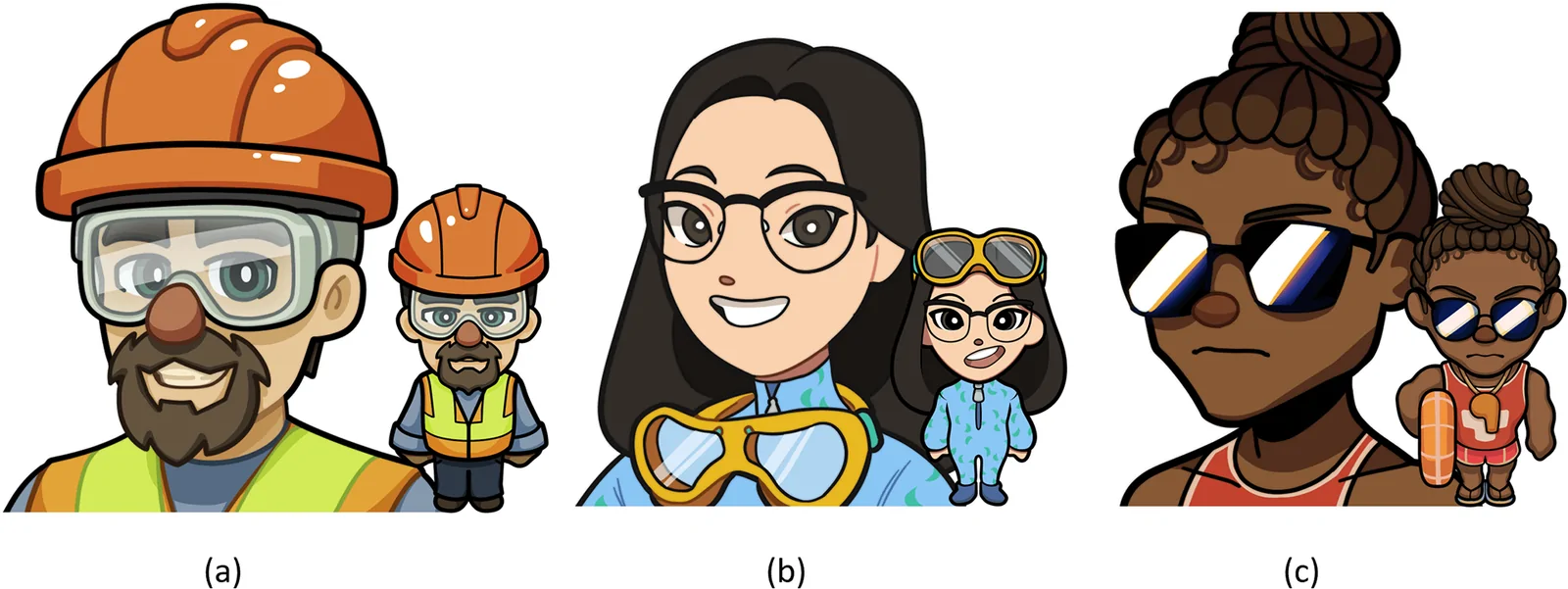
AI-powered non-player characters used in the endocrine level. Portrait and in-game sprite depictions of the endocrine level AI-powered NPCs. (**a**) Cooper, thyroid-family content specialist; (**b**) Josea, pancreas-family content specialist; and (**c**) Addy Stone, adrenal-family content specialist. Each character was available as a standard quest-related NPC and as a separate AI-enabled version. In the playable game, the AI-enabled version was identified with a blue AI-star indicator and was designed to answer open-ended scientific or medical questions related to its associated organ-family specialty.

**Figure 9.**
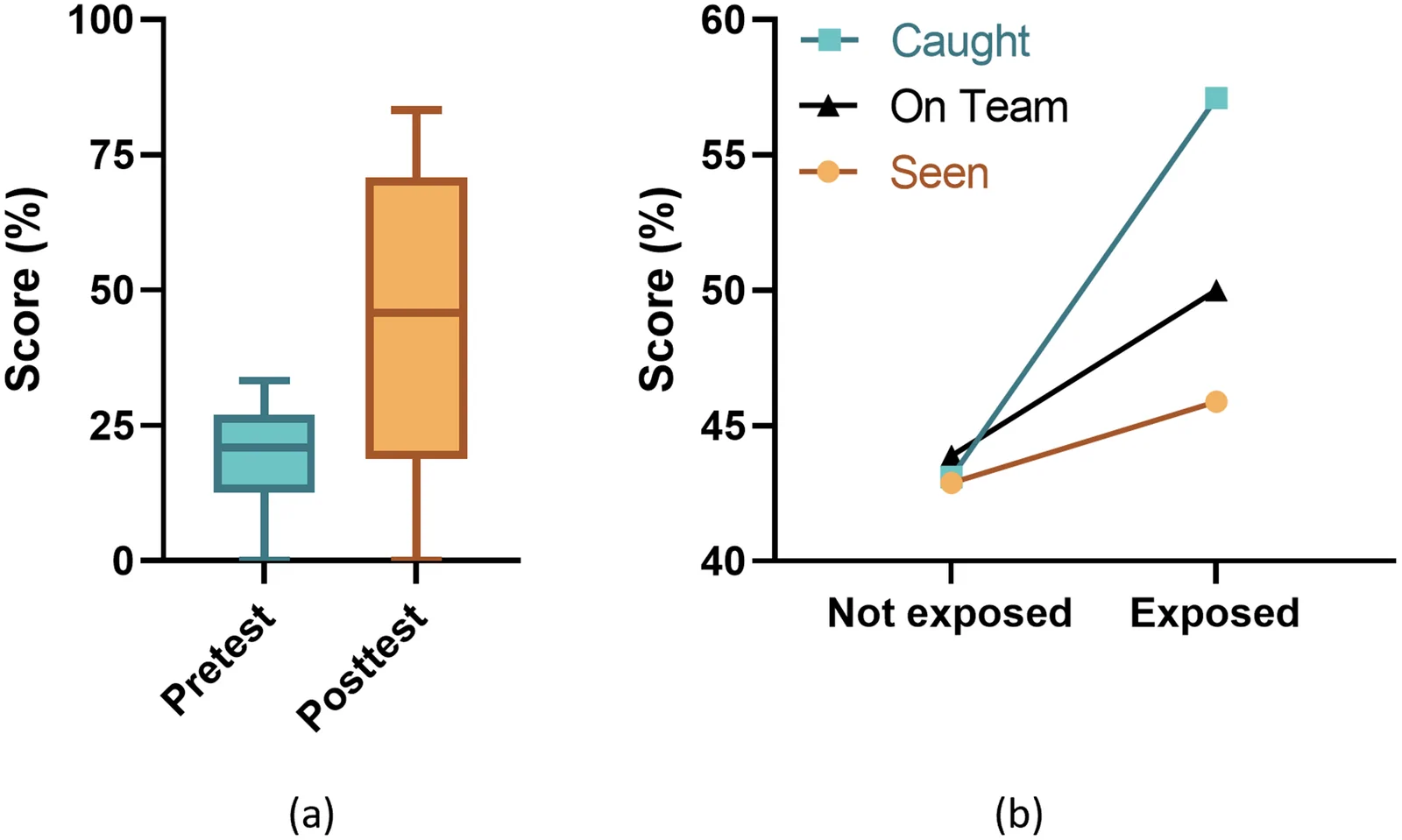
Preliminary endocrine level knowledge outcomes and exploratory Medimon exposure analysis. (**a**) Box plots show the percentage of correct responses on the 12-item endocrine level pretest and posttest among students who completed both assessments (*n* = 6). Boxes indicate the interquartile range, center lines indicate the median, and whiskers indicate the minimum and maximum observed values. (**b**) Exploratory posttest achievement scores for items associated with Medimon that students had seen, caught, or included on their final active team, compared with items associated with Medimon not exposed at each interaction level.

**Table 1.** Modified PXI player experience scores among high school Medimon playtest participants (*n* = 8). Scores are reported on a PXI-style scale ranging from −3 to +3, with 0 representing neutral and higher values indicating a more favorable experience. Favorable ratings were defined as participant-level construct means greater than 0.

Construct	Score, Mean ± SD	95% CI	Favorable Ratings, *n* (%)	FDR-Adjusted *q* Value
Meaning	0.75 ± 1.04	[−0.12, 1.62]	7 (87.5)	0.119
Curiosity	1.92 ± 0.83	[1.22, 2.61]	8 (100.0)	0.002
Mastery	0.79 ± 1.28	[−0.28, 1.86]	5 (62.5)	0.155
Autonomy	1.29 ± 1.39	[0.13, 2.45]	7 (87.5)	0.099
Immersion	0.46 ± 1.17	[−0.52, 1.44]	4 (50.0)	0.331
Progress Feedback	−0.12 ± 1.59	[−1.46, 1.21]	3 (37.5)	0.831
Audiovisual Appeal	2.04 ± 0.49	[1.64, 2.45]	8 (100.0)	<0.001
Challenge	1.17 ± 1.32	[0.07, 2.27]	6 (75.0)	0.099
Ease of Control	1.00 ± 1.64	[−0.37, 2.37]	5 (62.5)	0.155
Goals and Objectives	0.88 ± 1.14	[−0.07, 1.83]	6 (75.0)	0.119
Enjoyment †	1.67 ± 1.13	[0.72, 2.61]	7 (87.5)	0.016
AI Character Experience †	0.79 ± 1.08	[−0.11, 1.70]	5 (62.5)	0.119

† Supplemental project-specific scales rather than standard PXI constructs.

## Data Availability

Deidentified quantitative data supporting the findings of this study are available from the corresponding author upon reasonable request (tbland@uidaho.edu).

## Abbreviations

The following abbreviations are used in this manuscript:

AI: Artificial intelligence
NPC: Non-player character
LLM: Large language model
PXI: Player Experience Inventory
