# Supplementary material for: A Mixed-Methods Feasibility Pilot Study of Medimon for High School Endocrine Education
Source: Multimodal Technol Interact. Author manuscript; Available in PMC 2026 Oct 23. (PMC13690413; doi:10.3390/mti10080084)
Supplement: Supplementary file 1 [file NIHMS2203126-supplement-Supplemental.zip › Supplemental/Methods S1.pdf]

DOWNLOAD INSTRUCTIONS

1. Download the [Steam app](https://store.steampowered.com/) and create a free account if you don't already have one.
2. In the Steam app, click the **Add a Game** in the bottom left corner
3. Select **Activate a Product on Steam…** and enter the Steam key from your invite email.
4. Install the game and hit play

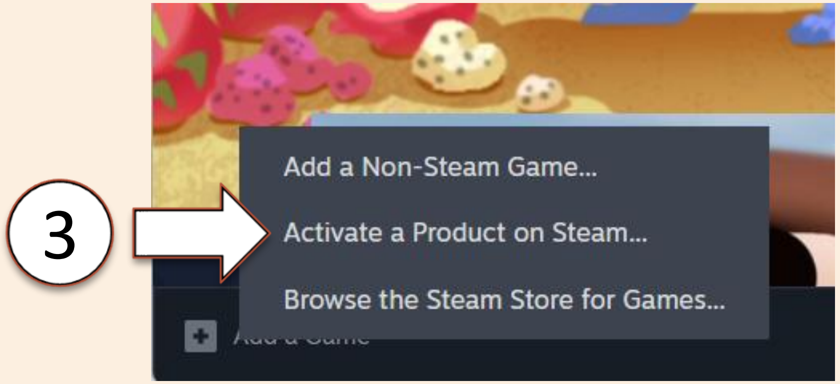

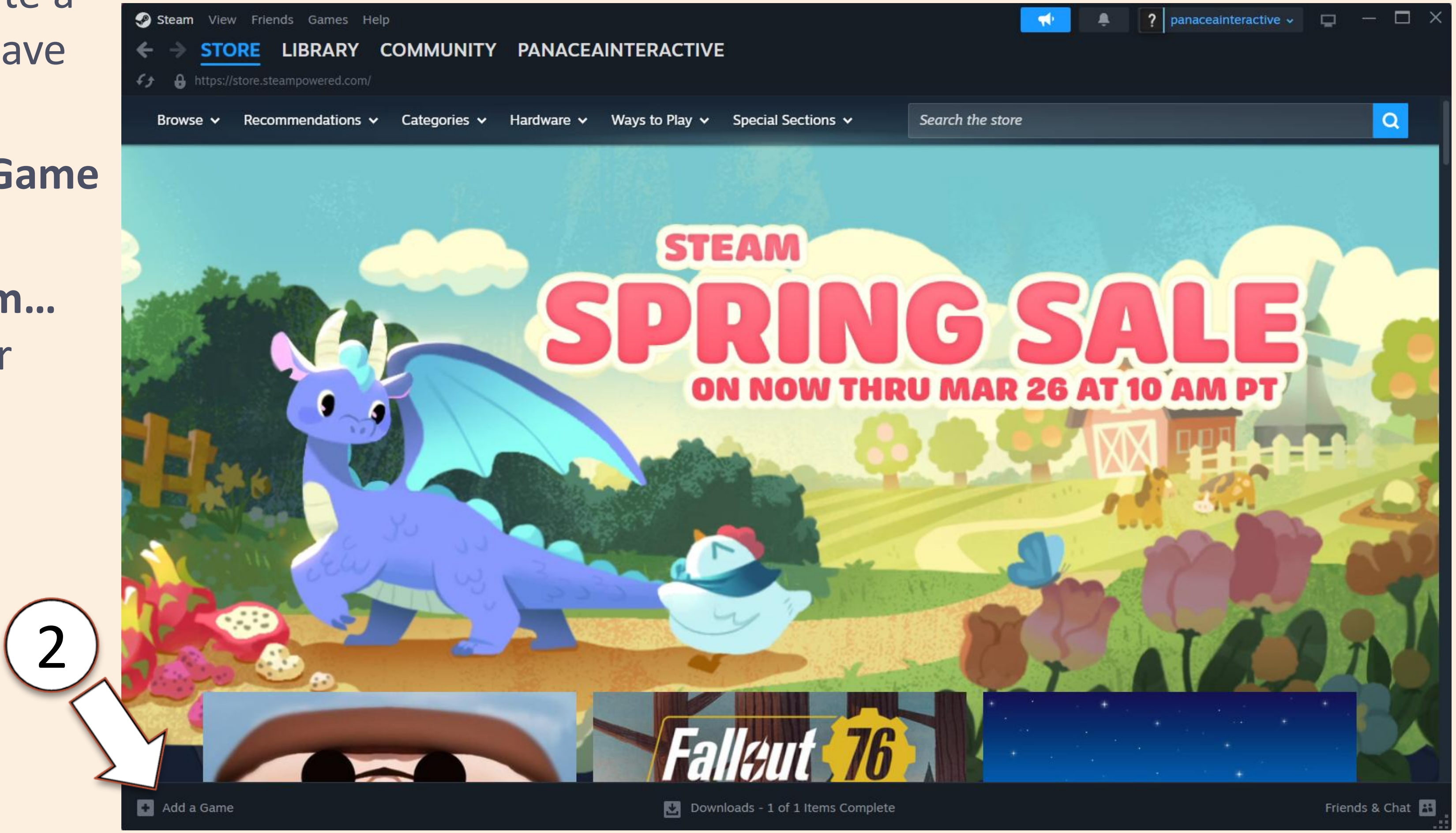

PLAYING THE GAME

- **Tutorial Video:**
  - This video give you an idea of how to play. This was built from an older version, so it will look a little different, but the game mechanics remain the same with the current game.

- **Goals:** Your main goal is to learn as much as you can about the Medimon.
  - **Complete quests** by interacting with NPCs (other characters) to progress through the game. This is how you unlock levels and progress through the game.
  - **Catch wild Medimon** and build up a strong team.
    - Defeating opponents gives your Medimon experience which is used to level up and make them stronger.
    - Visit the **medical tent** or **hospital** to heal your team. Each visit heals your entire team's health, but you need to use the correct treatment if you want to heal a diseased to a healthy version.
  - Interact with smart, **AI-powered NPCs** that embody specific medical specialties (more on the next slide)
    - **Note:** you will have to complete quests to unlock new sections which will provide you access to more of the AI-NPCs

SMART NPCS

- Some of the NPCs in the game are smart NPCs powered by AI which allows you to ask open ended questions.
  - Designed by the blue AI stars symbol
  - Designed to answer scientific/medical questions related to their specialty

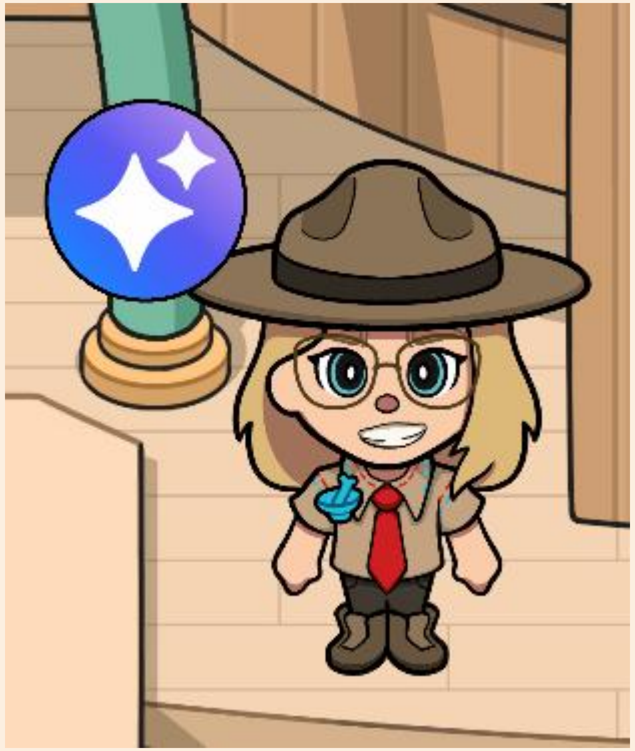

- Please feel free to ask these smart NPCs any scientific/medical question related to their specialty.
  - **Note:** They are only trained to provide scientific questions related to their specialty. They cannot answer how to perform actions inside the game like quests/battles.

AI-NPC INTERACTION GUIDELINES

The AI-powered smart NPCs in Medimon are designed for educational and gameplay purposes only. When interacting with the smart NPCs, please do not share:

- Personal identifying information (e.g., full name, phone number, address)
- Sensitive personal information
- Financial information
- Medical or health information about yourself or others
- Passwords or account information
- Please keep all interactions focused on educational content related to Medimon.

Smart NPC conversations are anonymously logged for research purposes as part of this study.

TYPE STRENGTH/WEAKNESS CHART

DEFENDING / ATTACKING	None	Normal	Structure	Endocrine	Immune	Circulatory	Fluids	Gut	Neural	Senses	Reproduction	Offence Avg
None	1	1	1	1	1	1	1	1	1	1	1	1.00
Normal	1	1	0.5	1	0.5	1	1	1	1	1	1	0.89
Structure	1	1	0.5	0.5	0.5	2	1	2	1	2	0.5	1.11
Endocrine	1	1	2	1	0.5	2	1	1	1	2	0.5	1.22
Immune	1	1	0.5	1	1	0.5	0.5	2	0.5	1	0	0.78
Circulatory	1	1	0.5	2	2	1	2	1	0.5	1	1	1.22
Fluids	1	1	1	1	0.5	0	0.5	0.5	0.5	2	2	0.89
Gut	1	1	1	1	0.5	1	2	1	2	2	0.5	1.22
Neural	1	1	2	1	1	2	1	0.5	1	0.5	0.5	1.06
Senses	1	1	1	0.5	0.5	1	1	1	2	1	2	1.11
Reproduction	1	1	2	2	2	1	1	1	1	0.5	2	1.39
Defence Avg	1.00	1.00	1.10	1.10	0.90	1.15	1.10	1.10	1.05	1.30	1.00	

DISCLAIMER

This is a very early version of the game. Yes there will be bugs, but we hope there are currently none bad enough to break the game for you.

If you would like to help with the development and report any bugs that you find in the game, you can learn more at our website www.medimon.games and join our discord.

We greatly appreciate your help, and we hope you enjoy Medimon!!!
