# Supplementary material for: A Mixed-Methods Feasibility Pilot Study of Medimon for High School Endocrine Education
Source: Multimodal Technol Interact. Author manuscript; Available in PMC 2026 Oct 23. (PMC13690413; doi:10.3390/mti10080084)
Supplement: Supplementary file 1 [file NIHMS2203126-supplement-Supplemental.zip › Supplemental/Methods S3.docx]

Level 2 Pre/posttest

1. **Which of the following is characteristic of pancreatitis?**
   a. Increased pancreatic function
   b. Beta cell growth
   c. Autodigestion of the pancreas
   d. I have no idea
2. **Which hormone from the adrenal cortex is released during a stress response?**
   a. Insulin
   b. Melatonin
   c. Cortisol
   d. I have no idea
3. **Which of the following organs is involved in digestion?**
   a. Thyroid gland
   b. Adrenal glands
   c. Pancreas
   d. I have no idea
4. **Which of the following can cause Cushing syndrome?**
   a. Too little insulin
   b. GC (glucocorticoid) medications
   c. Too much thyroid hormone
   d. I have no idea
5. **Calcitonin released by C-cells reduces the level of what ion in the blood?**
   a. Sodium
   b. Potassium
   c. Calcium
   d. I have no idea
6. **What response is the adrenal medulla most involved in?**
   a. Fight-or-flight response
   b. Digestion response
   c. Sleep response
   d. I have no idea
7. **What happens in Hashimoto disease?**
   a. The pancreas makes too much insulin
   b. Antibodies attack and damage the thyroid gland
   c. The adrenal gland releases too much cortisol
   d. I have no idea
8. **What hormones are produced by the thyroid gland?**
   a. T3/T4
   b. Insulin/glucagon
   c. Cortisol/aldosterone
   d. I have no idea
9. **In what state are alpha cells of the pancreas active?**
   a. Fed state
   b. Fasted/Starved state
   c. Sleepy state
   d. I have no idea
10. **What cells are destroyed in Type 1 Diabetes?**
    a. Beta cells of the pancreas
    b. Alpha cells of the pancreas
    c. Hepatocytes of the liver
    d. I have no idea
11. **In Addison disease, which organ is not working properly?**
    a. Adrenal gland
    b. Pancreas
    c. Thyroid gland
    d. I have no idea
12. **Which organ becomes overactive in Graves disease?**
    a. Adrenal gland
    b. Pancreas
    c. Thyroid gland
    d. I have no idea
